# Tool Wear Effect on Surface Integrity in AISI 1045 Steel Dry Turning

**DOI:** 10.3390/ma15062031

**Published:** 2022-03-09

**Authors:** Laurence Colares Magalhães, Gabriel Catarino Carlesso, Luis Norberto López de Lacalle, Marcelo Tramontin Souza, Fabiana de Oliveira Palheta, Cristiano Binder

**Affiliations:** 1 Department of Industrial Technology, Federal University of Espírito Santo (UFES), Vitória 29075-910, Brazil; gabriel.carlesso@ufes.edu.br; 2 Department of Mechanical Engineering, CFAA—University of the Basque Country (UPV/EHU), Zamudio Technologic Park, 48170 Bilbao, Spain; 3 Department of Exact and Technological Sciences, Santa Cruz State University (UESC), Ilhéus 45662-900, Brazil; mtsouza@uesc.br; 4 Department of Mechanical Engineering, Federal University of Santa Catarina (UFSC), Florianópolis 88040-900, Brazil; fabiana.palheta@labmat.ufsc.br (F.d.O.P.); cristiano.binder@ufsc.br (C.B.)

**Keywords:** dry turning, surface integrity, tool wear

## Abstract

In the present work, the surface integrity and flank wear of uncoated cermet inserts in dry turning of AISI 1045 steel were evaluated. Three-dimensional techniques were used to assess the surface roughness. Previously, finite element analysis was carried out to predict the cutting forces and heat distribution in the chip formation region. Cutting speed and feed were the parameters varied in the experiments. Feed is decisive in the final quality of the turned surface and cutting speed had little influence on this aspect. The surface was significantly damaged with the progression of the insert flank wear. Considering an average flank wear *VB_B_* of 0.1 mm, a tool life of 35 min was achieved using a cutting speed of 175 m/min, and of 23 min for a cutting speed of 275 m/min. Abrasive wear was predominant during the experiments. No microstructure defects were observed, as well as crack propagation or accentuated deformations near the machined surface region. Therefore, the dry turning of 1045 steel with cermet inserts route has proven extremely viable from the standpoints of tool life, surface integrity, chip formation, and sustainability.

## 1. Introduction

Machining industries are interested in high material removal rates and high product quality by using the greatest cutting speed and feed rates to achieve better productivity. It becomes extremely tough to attain these properties as the high cutting temperature produced in the cutting zone causes premature failure of the cutting tools, which results in poor dimensional accuracy. It also weakens the surface integrity of the product by inducing tensile residual stresses and surface and sub-surface microcracks in addition to rapid oxidation and corrosion [1]. Residual stresses are always a serious concern regarding the fatigue life of components. 

The main functions of cooling lubricants are to reduce heat generation by reducing friction and to eliminate the exertion of unacceptable influences on the structure of the subsurface layer of the workpiece by absorbing and removing heat from the cutting area. In cutting operations, cooling lubricants also have an important transport function for the chips which have to be removed. Thus, efficient lubrication systems enable high-performance operations in practice [2].

In dry machining operations, there is no coolant. This means that there will be more friction and adhesion between the tool and workpiece. Tools and workpieces are subjected to greater thermal loads. This may result in higher levels of tool wear, e.g., in increased crater formation when steel materials are machined using uncoated carbides. However, the dry cutting may also show positive effects such as a reduction in thermal shock and thus in the formation of comb cracks when parts are machined in interrupted mode with carbides or cermets. Higher machining temperatures influence chip formation. This may result in both ribbon chips and snarl chips [2]. 

The usage of cooling lubricants imposes environmental problems due to their chemical breakdown at high temperatures and it contaminates water and soil during mass disposal. It also imposes a high cost for the setup of a coolant system, as it has to be stored, pumped, filtered, and recycled when it is used. It also has adverse effects on the parts of the machine tool and workpiece causing corrosion, which leads to its failure. Besides, attention to health precautions cannot be neglected [1,2].

Recent advances in cutting tools materials have successfully enabled the use of dry cutting or MQL (minimum quantity of lubrication), notably in steels and alloys. In some cases, the surface quality is the same or better when dry cutting is used. Moreover, depending on the selected cutting parameters, tool wear in dry cutting is not that different when MQL is used [3]. 

Considering tool materials for dry cutting, cermets stand out due to their high hot hardness, low reactivity with steels and other metals, and especially low thermal conductivity compared with cemented carbide [4]. So, Klocke [5] compares the properties of the principal constituents of a typical cemented carbide (WC-6CO) with cermet (titanium carbide, TiC—based) inserts. Tungsten carbide (WC) has an average microhardness of 1580 HV (HV30) and thermal conductivity of 80 W/(m.K), while for TiC, these values are 3100 HV (HV0.05) and 33 W/(m.K), respectively. Furthermore, some features (such as wear resistance, edge strength, and sharpness, ability to cut at high speeds employing moderate feeds and depths of cut, and high quality of the machined surface) are responsible for the superior cutting performance of cermets [6]. The properties, performance, and cost, comparable with cemented carbides, make cermet tools a competing alternative to semi-finish and finish machining of steels and cast irons [7].

Some works have investigated the turning of alloy steels under different cooling conditions employing different cutting materials. REIS et al. [8] investigated the cutting performance and wear behavior of single-coated cermet and multilayer-coated carbide tools with distinct chip breaker geometries when dry turning AISI 4340 steel. With regard to tool wear, the coated cermet tool showed the lowest values for maximum flank wear (*VB_Bmax_*) compared with the cemented carbide tool. Crater wear was also lower on the cermet insert. Adhesive and abrasive wear mechanisms were observed, respectively, in the rake and flank faces of both tools.

Maruda et al. [9] evaluated the cooling effect under minimum quantity cooling lubrication and dry cutting on structural changes and microhardness of the ferritic–pearlitic AISI 1045 steel after turning. The tests showed that cooling of the cutting zone under minimum quantity cooling lubrication (MQL) condition decreased the depth of the hardened layer compared with dry cutting by approximately 40% for both pearlite and ferrite phase structures. The microhardness of the perlite phase on the top surface was 430 (HV 0.05) on dry condition and 340 HV on MQL condition. As a result of cooling being applied to the cutting zone using the MQL method, the average diameter of ferrite grains has been decreased in the entire surface area compared with dry cutting.

Sarjana et al. [10] evaluated uncoated cermet tools made of titanium carbonitride (TiCN) as core particles, (Ti, Nb, and W) (C, N) for the second hard phase, and a W-rich Co binder and a PVD-coated (TiCN/TiN) tool to the turning of high-strength low-alloy steel AISI 4340 (hardened to 50 HRC). The results of the study show that both cermet tools can be recommended to support the work of coated cemented carbide, in particular, to finish the turning of the hardened steel. The optimum cutting condition at cutting speed of 120 m/min, feed of 0.1 mm/rev, and depth of cut of 0.2 mm give the best result in terms of productivity. The uncoated tool showed better results in terms of productivity and the PVD-coated tool showed better results in terms of surface quality. Flank wear was the dominant wear and failure mode of both cermet cutting tools when assigned to finish hard turning. Besides, chipping was also observed, and this wear mode started after a certain limit of flank wear progression (after *VB_B_* ~ 125 μm for uncoated and after *VB_B_* ~ 100 μm for PVD-coated).

Yang et al. [11] studied the flank wear mechanism of (Ti,W)C–Mo2C–Co cermets during dry turning of a high carbon alloy steel hardened to 62 HRC. Considering the average flank wear of *VB_B_* = 0.3 mm, the tool life was about 33 min to a cutting speed of 180 m/min. Tool life decreased around 40% when the cutting speed was raised to 280 m/min and 75% when the cutting speed was raised to 450 m/min. At lower cutting speeds, only abrasive wear was noticed and adhesion was observed at higher cutting speeds. Tool life decreased 75% when the depth of cut was raised from 0.22 to 0.5 mm, and 22% when the feed was raised from 0.08 to 0.24 mm/rev. Depth of cut was the most significant parameter influencing diffusive wearing. Four wear mechanisms, including abrasive wearing, adhesive wearing, diffusive wearing, and oxidative wearing, aggravate the flank wear of (Ti, W) C-based cermet inserts differently when varying cutting speed, feed rate, or depth of cut.

Das et al. [12] compared the performance of uncoated carbide with coated cermet inserts in dry turning of AISI 4340 steel (hardened at 48 HRC). The authors analyzed the part surface temperature, cutting forces, and flank wear. Cutting forces and tool wear were lower with the use of cermets.

Grzesik [13] studied the influence of tool wear on surface roughness in the hard turning of AISI 5140 steel with mixed ceramic wiper inserts. The author draws attention to the notch wear on the secondary flank as an aggravating factor in the roughness of the turned surface. 

Sampaio et al. [3] analyzed the wear process of PCBN (polycrystalline cubic boron nitride) cutting tools in the hard turning of hardened SAE 1045 steel using MQL and compared it with dry machining. In terms of surface roughness, dry cutting presents better performance. In dry conditions, for a cutting speed of 150 m/min with a depth of cut set at 0.2 mm, the tool flank shows severe damage with chipping of the tool edge because the crater wears extended up to the flank region. Adhesion, abrasion, and plastic deformation could be observed as wear modes in the dry condition. In general, the MQL condition can reduce the crater and flank wear and white layer thickness. 

Abbas et al. [14] compared the effectiveness of using dry, flood, and MQL methods when turning the AISI 1045 steel. Better surface roughness and power consumption performance were achieved at MQL conditions. However, the lowest machining costs were noticed in dry-cutting conditions.

Magalhães et al. [15] evaluated the wear of coated cermet inserts in turning 1020 steel applying coolant. The results showed that for a cutting speed of 290 m/min and considering the average flank wear of *VB_B_* = 0.3 mm, the tool life is 113 min, which demonstrates the feasibility and success of this tool material for general industrial processes. The same author investigated the influence of the feed and tool geometry on the surface roughness in the high-speed turning of 4340 steels. It was found that the feed is a capital factor in this sense and the corner radius of the insert has little influence in this aspect. Arithmetical mean height values of *Ra* = 0.35 µm were obtained when the best parameters were selected, i.e., smaller feeds and larger corner radii [16]. Zhang and Wu [17] studied the chip control in the dry turning of hardened AISI 1045 steel (52~58 HRC). Negative CBN inserts were used in the experiments. The studies were carried out from two perspectives: conventional and high-speed cutting. The results showed that continuous ribbon chips can be produced at a low cutting speed of 110 m/min, and the chip thickness is relatively uniform. Chips demonstrate saw-tooth morphology at a cutting speed of 276 m/min. When high cutting speeds were used (414 and 552 m/min), serrated chipping took place. These chips were easily broken into short chips of 1~3 cm lengths. The surface roughness obtained at high speeds ranged from 0.63 µm to 1.6 µm, proving that hard turning is feasible to be implemented in industrial applications since it can reach the same level as that achieved by the grinding process in terms of surface roughness.

Kumar et al. [18] investigated the performance of TiAlCrN-coated tungsten carbide tools during AISI 1045 steel dry turning. The application of the TiAlCrN coating caused a significant reduction in the coefficient of friction, which resulted in a reduction in tool stresses. Besides, the coating acts as a thermal barrier for the substrate, it makes the removal of the hot chip faster, thus reducing the time of contact with the workpiece.

Finally, various authors have proposed alternative cooling methods in machining, with CO_2_ cryogenics being the most promising one because of its low cost and maximum cooling action. Amigo et al. [19] obtained good results in hard turning. Other authors such as Suárez et al. [20] proposed the use of high pressure with emulsions coolants instead of conventional pressure values. Even with the good results of those works, dry turning will also protect the health and safety of the workers.

In this work, the dry turning of AISI 1045 steel was studied. Uncoated cermet inserts were used. Feed and cutting speed were varied and surface integrity and tool wear were evaluated. Finite element analysis was performed to understand chip formation, heat generated in the cutting zone, and cutting forces. Within this context, it is expected to contribute to a more sustainable machining process, verifying if the combination of the use of modern cermets in dry turning in the processing of medium carbon steels can be a viable route from the point of view of surface quality, tool life, and microstructure.

## 2. Materials and Methods

### 2.1. Experimental Procedure

**Figure 1:**
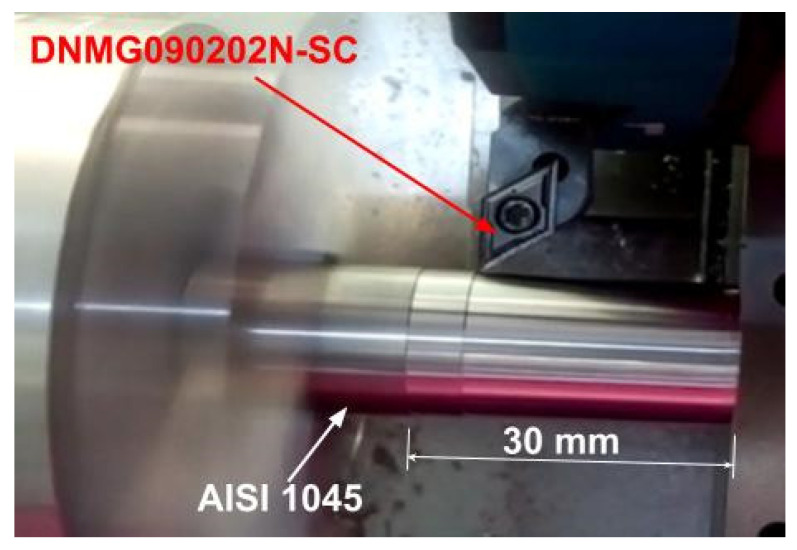
Part and tool fixture scheme during performed tests.

External cylindrical dry turning was performed on AISI 1045 Steel (wt%—0.48 C, 0.73 Mn, 0.25 Si, 0.016 P, 0.05 S, 0.02 Al, 0.02 Cr, and 0.01 Ni—manufacturer datasheet) round bars with 30 mm diameter and 55 mm length in a Boxford^®^ CNC lathe model 160 VMCi (Halifax, UK) (0.5 KW power and 3200 maximum rpm). The 1045 steel was machined in its state of supply and presented a hardness of 248 HB; its mechanical properties are shown in Table 1. A three-jaw air chuck was used to clamp the parts. The positive (6° rake angle) uncoated cermet inserts grade T1200A geometry DNMG090202N-SC and SDACR062B tool holder supplied by Sumitomo Tools (Osaka, Japan) were used on experiments (Figure 1). According to the manufacturer, the inserts present a tough composite phase of coarse grains, a W-rich tough hard phase, and a fine TiCN grain phase in the binder phase.

Two values of cutting speed (*Vc*): 175 m/min and 275 m/min, and four values of feed (f): 0.025 mm/rev, 0.05 mm/rev, 0.075 mm/rev, and 0.01 mm/rev were varied during the tests. Depth of cut (*doc*) was kept constant at 0.2 mm (100% of tool corner radius), as shown in Table 2. Before each test, an initial preparation pass with a dedicated tool was performed to uniform the surface. Each test was repeated twice and a new cutting edge was used for each test. Divergence was lower than 5% in each condition. 

### 2.2. Finite Elements Analysis (FEA)

Finite element analysis was performed to predict cutting forces, heat flow in the cutting zone, and chip formation. AdvantEdge V7.1 software (2015, Minneapolis, MN, USA) was used to simulate the orthogonal cut for the two cutting speeds (175 m/min and 275 m/min) and the lower and higher feed levels (0.025 mm/rev and 0.1 mm/rev). 

The friction coefficient was established according to tool geometry and the material software default of the general ceramic tool. AdvantEdge simplifies the friction coefficient as defined by Coulomb friction in the following Equation (1):

(1) Ff=μ×Fn

where *Fn* is the normal force exerted between the surfaces, µ is the coefficient of friction, and *Ff* is the resulting force due to friction. AISI 1045 steel data were customer-defined with Table 1 data and equivalent chemical composition. The workpiece meshing was defined as a maximum and minimum element size of 0.1 mm and 0.02 mm, respectively. The mesh refinement and coarsening factor were kept as defaults, 2 and 6, respectively. The maximum number of nodes was adjusted to 2400. Thirty output frames were adopted. Tecplot 360 R2 software (2020, Bellevue, WA, USA) was used for data analysis and treatment. A Carl Zeiss Discovery V12 stereomicroscope (Oberkochen, German) equipped with AxioCam 305 (Oberkochen, German) and AxioVision V4.7 software (2008, Jena, Germany) was used to measure chip thickness and compare these results with FEA analysis.

### 2.3. Surface Roughness

At first, the topography of surfaces machined with new inserts was evaluated using white light interferometry. With this technique, better visualization and evaluation of the surface integrity is possible. It was employed with the New View 7300 interferometer from Zygo (Middlefield, CT, USA) and Leica (Wetzlar, HE, German). A scan rate of 100 µm/s and a magnification of 20× were used. The accuracy of the former equipment is less than 0.75%, the lateral resolution from 0.36 to 9.5 nm, and the vertical resolution 0.1 nm.

In the second stage, the surface roughness was evaluated at intervals of 150 mm of machined length together with the tool wear evaluation, so a roughness × flank wear curve could be plotted. At this stage, a Taylor Hobson Surtronic 25 roughness meter (Leicester, England) was employed. A cut-off of 0.25 mm according to ISO 4288 [21] was established. The results are an average of three measurements performed on each sample. The results were compared with theoretical roughness (*h*) following Equation (2), where *Re* is the insert corner radius. For the *Ra* parameter, this may be written as *h*/4 [22].

(2) $$h=\frac{f^{2}}{8Re}$$

### 2.4. Tool Life and Tool Wear

The tool life tests were performed using an end-of-life criterion based on an average width of the flank wear land *VB_B_* = 0.1 mm based on the ISO 3685 [23] standard. An average of three measurements were taken. Tool wear was measured at intervals of 150 mm of machined length. The flank wear was measured using a Carl Zeiss Discovery V12 stereomicroscope equipped with AxioCam 305 (Oberkochen, BW, Germany) and AxioVision V4.7 software (2008, Jena, Germany) to acquire and process digital images. 

### 2.5. Microstructure and Microhardness Evaluation

**Figure 2:**
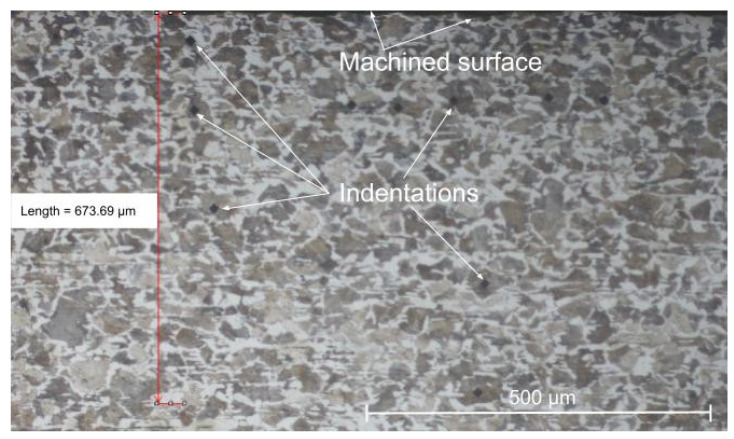
Microhardness indentations on the pearlite phase in a sample machined at *Vc* = 275 m/min and f = 0.1 mm/rev.

To evaluate the surface integrity under the machined surface, microhardness tests on each microconstituent of the material (pearlite—dark phase and ferrite—white phase), were performed. Samples were cut and embedded in Bakelite and then sanded and polished with alumina suspensions with granulation of 1 µm. The metallographic sections were etched using a 2% nitric acid solution in ethanol (Nital) during 10 s. A Vickers indenter with a load of 25 g during 12 s was used in the test for all the measurements which were performed beneath the machined surface in depths of 25 µm, 125 µm, 225 µm, 325 µm, and 425 µm, as shown in Figure 2 details. Three measurements were performed at nearby points. A Shimadzu HMV-G 20ST hardness tester (Kyoto, Japan) was used to perform microhardness evaluation. The Nikon Eclipse MA200 microscope (Kyoto, Japan) was used to analyze material microstructure after the machining process.

## 3. Results

### 3.1. FEA Analysis Results

**Figure 3:**
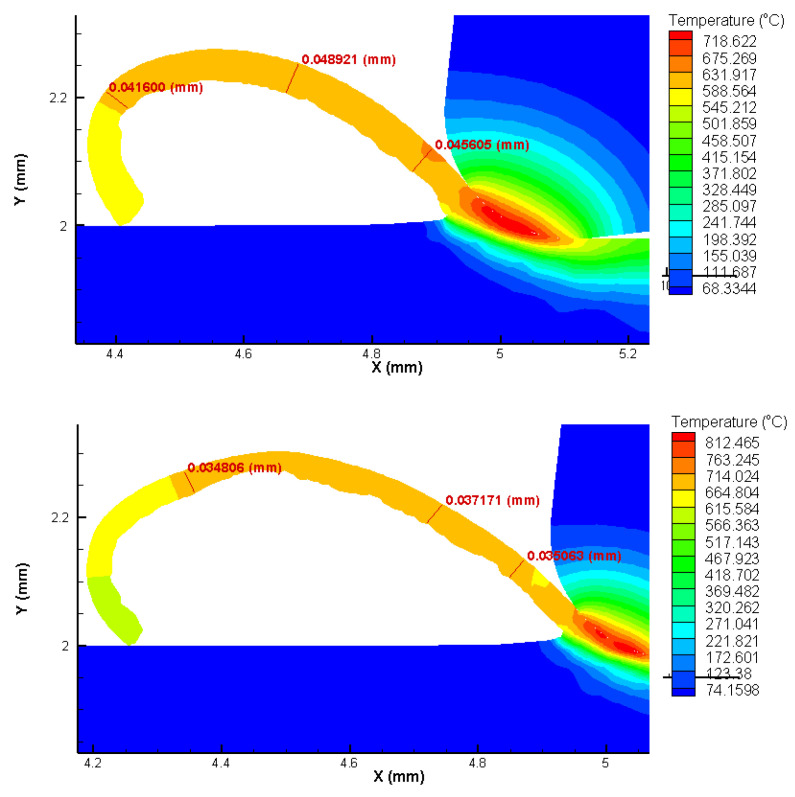
Heat distribution and chip thickness in Finite Elements Analysis (FEA) for *f* = 0.025 mm/rev. *Vc* = 175 m/min (**top**) and *Vc* = 275 m/min (**below**).

**Figure 4:**
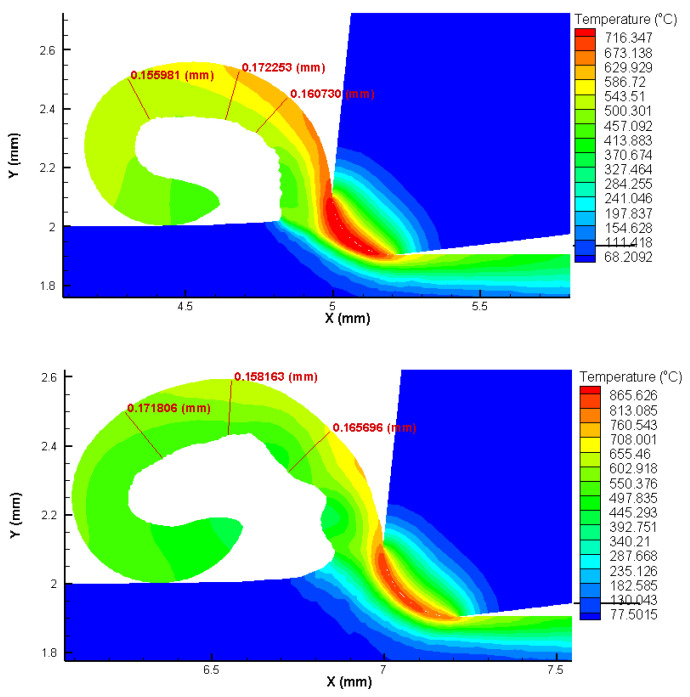
Heat distribution and chip thickness in FEA analysis for *f* = 0.1 mm/rev. *Vc* = 175 m/min (**top**) and *Vc* = 275 m/min (**below**).

The FEA analysis results show that the temperature in the tool part contact zone can exceed 860 °C for the highest cutting speed and feed settings used in the experiments, while 17% lower temperature values are found for the lowest cutting speed and feed settings used. The chips are predominantly in the form of twisted ribbons of the continuous type, for all test configurations (Figure 3 and Figure 4). 

**Figure 5:**
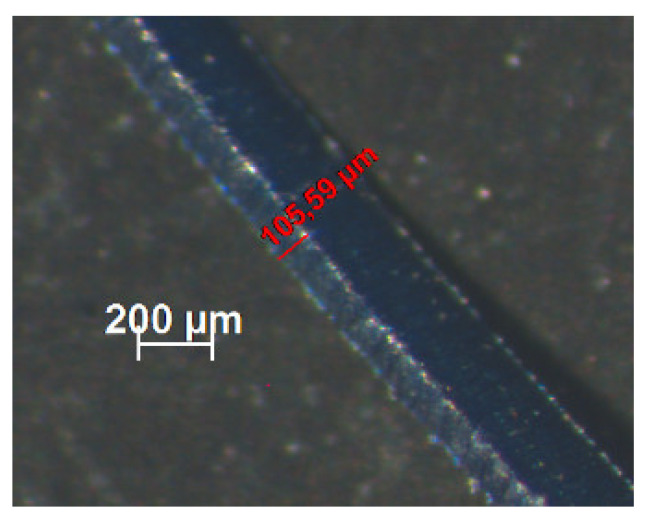
Chip thickness under optical microscope measurements for *Vc* = 175 m/min and *f* = 0.1 mm/rev.

**Figure 6:**
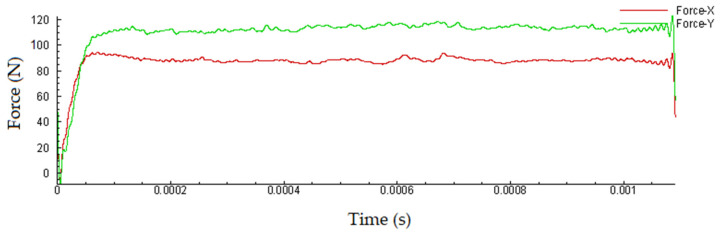
Cutting forces predicted by FEA analysis (filtered of numerical noise) for *f* = 0.1 mm/min and *Vc* = 275 m/min.

It is possible to verify a good agreement between the chip thickness values estimated by FEA and measured through microscopy by analyzing Table 3 and Figure 5. The smaller the feed, the smaller the chip shear angle. As expected, the cutting force values increase with increasing feed, however, the cutting forces estimated by the FEA analysis did not exceed 120 N in any setting of parameters used (Figure 6). The good concordance of the chips obtained with those estimated by FEA analysis, in terms of shape and thickness, shows that this technique has good reliability for the results presented and discussed. 

### 3.2. Surface Roughness and Tool Wear 

**Figure 7:**
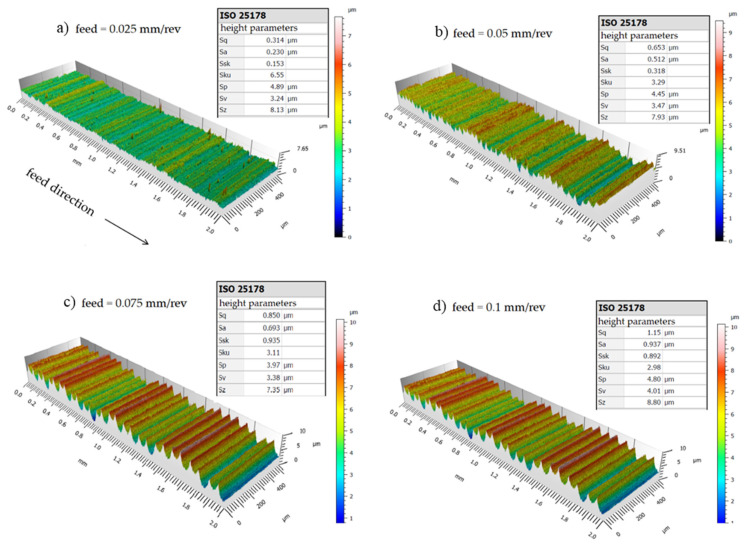
3D surface roughness for *Vc* = 175 m/min with different feeds. (**a**) 0.025 mm/rev, (**b**) 0.05 mm/rev, (**c**) 0.075 mm/rev, (**d**) 0.1 mm/rev.

**Figure 8:**
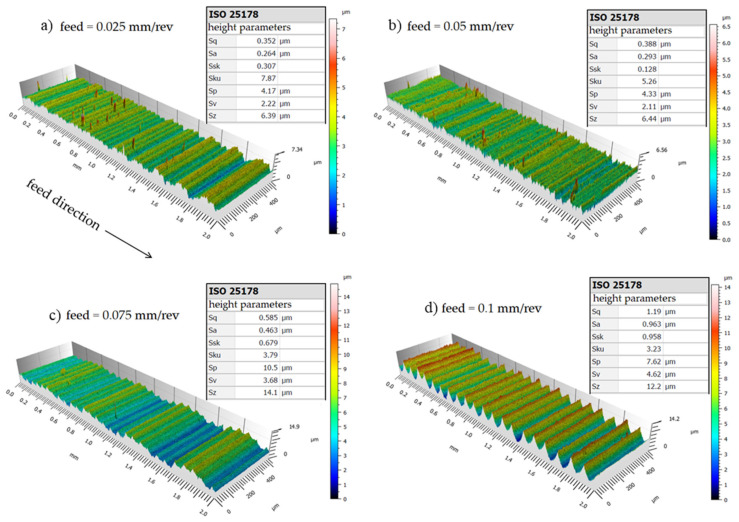
3D surface roughness for *Vc* = 275 m/min with different feeds. (**a**) 0.025 mm/rev, (**b**) 0.05 mm/rev, (**c**) 0.075 mm/rev, (**d**) 0.1 mm/rev.

**Figure 9:**
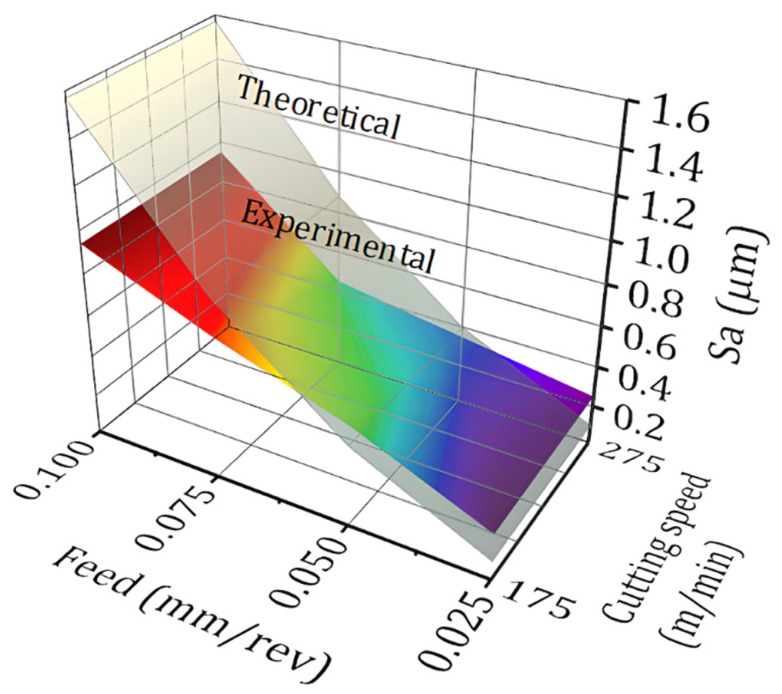
Arithmetic mean height (*Sa*) as a function of feed and cutting speed (new cutting edge).

The topography of the machined surface, as a function of feed, using a new cutting edge, is shown in Figure 7 and Figure 8 for cutting speeds of 175 m/min and 275 m/min, respectively, and the results are summarized in the graph shown in Figure 9. It is easy to see the effect of increased feed on machined surface degradation. For a feed of 0.025 mm/rev, an arithmetical mean height value (*Sa)* of 0.23 µm is reached, denoting a superfinishing surface state. This value reaches the 0.94 µm *Sa* mark, for a feed of 0.1 mm/rev. Considering the maximum height *Sz* parameter, the values are *Sz* = 8.13 µm and *Sz* = 8.8 µm, respectively, when the cutting speed is 175 m/min. The *Sa* parameter expands the profile (line roughness) three-dimensionally. It represents the arithmetic mean of the absolute ordinate Z (x,y) within the evaluation area. This is one of the most widely used parameters providing stable results since it is not significantly influenced by scratches, contamination, and measurement noise. The *Sz* parameter expands the profile (line roughness) parameter *Rz* three-dimensionally. The maximum height *Sz* is equivalent to the sum of maximum peak height *Sp* and maximum valley depth [24].

By increasing the cutting speed to 275 m/min, an arithmetical mean height value *Sa* = 0.26 µm is achieved, also denoting a superfinishing surface state. This value reaches the *Sa =* 0.96 µm mark, for a feed of 0.1 mm/rev. Considering the maximum height *Sz* parameter, the values are *Sz* = 6.4 µm and *Sz* = 12.2 µm, respectively. It is thus possible to establish that the cutting speed has little influence on the final roughness, however, if the *Sz* parameter is mandatory, it is better to use the combination of high cutting speed (275 m/min) and low feed (0.025 mm/rev). Nonetheless, it is worth considering that although frequently used, this parameter is significantly influenced by scratches, contamination, and measurement noise due to its utilization of peak values [24].

**Figure 10:**
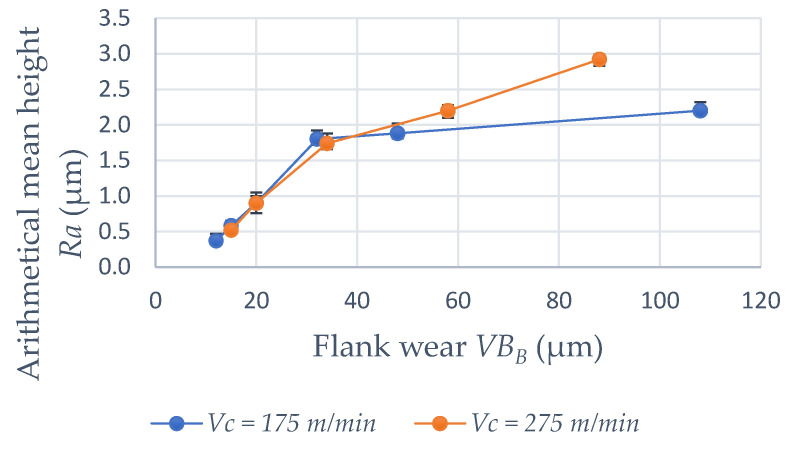
Deterioration of surface roughness with increased flank wear.

**Figure 11:**
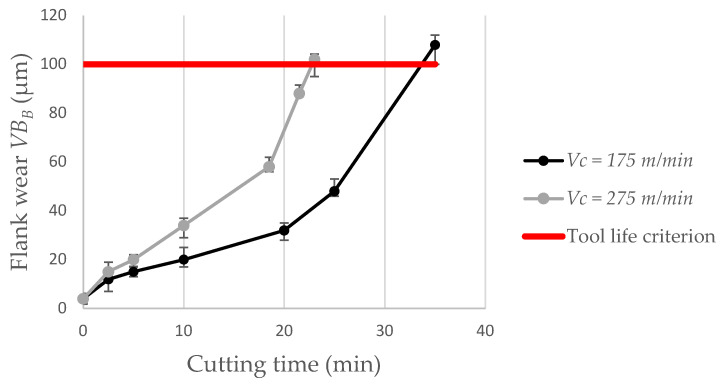
Tool life curves for the cermet insert on 1045 steel dry turning.

**Figure 12:**
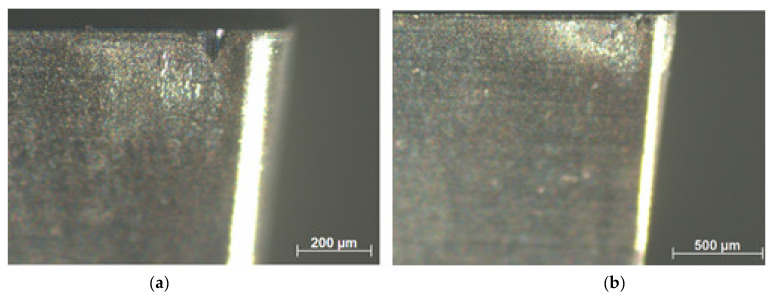
Flank wear in tool life criterion: (**a**) *Vc* = 175 m/min; (**b**) *Vc* = 275 m/min.

Surface roughness was also evaluated in terms of the degree of flank wear of the insert. These results are shown in Figure 10. In these tests, the feed was set at 0.025 mm/rev keeping the depth of cut at 0.2 mm. For a cutting speed of 175 m/min, the arithmetic mean height *Sa* is 0.23 µm for a new cutting edge and rises to *Ra* = 2.2 µm when the wear on the primary flank reaches *VB_B_* = 0.1 mm. When the cutting speed is 275 m/min, the roughness when the insert reaches the end of life criterion is *Ra* = 2.92 µm. Thus, it is important to realize that when it comes to a worn insert, a higher cutting speed leads to greater damage to the machined surface. Surface degradation with increased flank wear is already expected since with wear the friction in the tool part pair is greater, increasing the generated heat and cutting forces. Flank wear on the secondary edge is also of great importance for this significant increase in surface roughness, since, in fact, it is this tool geometry that makes the last contact in engagement with the machined surface [25]. Figure 11 shows the tool life curve for the cermet insert on the selected parameters and Figure 12 shows the wear on the primary flanks for the inserts when reaching the established end-of-life criterion. For a cutting speed of 175 m/min, a 35 min tool life is reached and this value is 23 min when a cutting speed of 275 m/min is used.

### 3.3. Microhardness and Microstructure 

**Figure 13:**
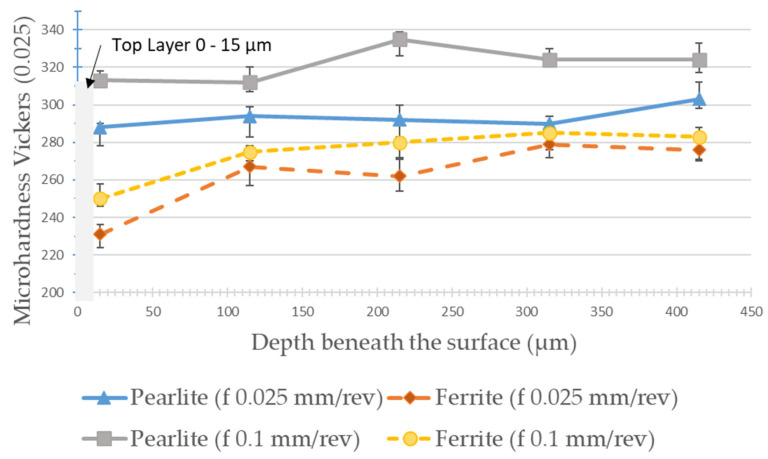
Microhardness from machined surface for *Vc* = 175 m/min on pearlite and ferrite phases with different feeds.

**Figure 14:**
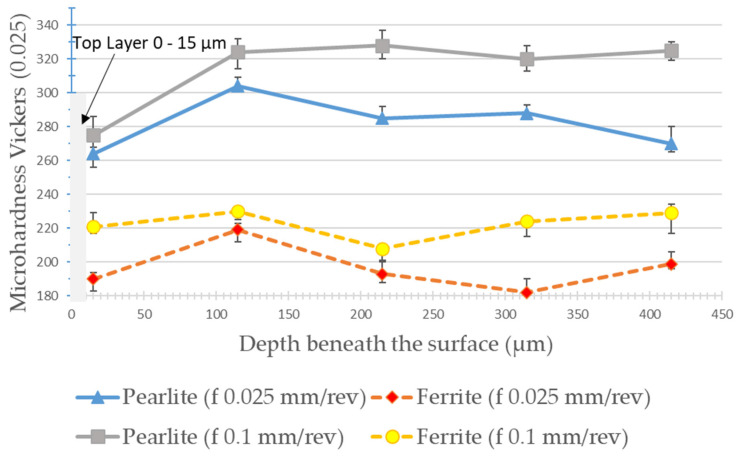
Microhardness from machined surface at *Vc* = 275 m/min on pearlite and ferrite phases at different feeds.

Figure 13 and Figure 14 show the results of microhardness, from the machined surface, in the micro constituents of the material (pearlite and ferrite) for the lowest and highest feed values used in the experiments and for the cutting speeds of 175 m/min and 275 m/min, respectively. For the cutting speed of 175 m/min, it is possible to observe that, in the ferrite phase, the hardness is reduced close to the machined surface (25 µm) reaching values in the order of 230 HV. From a depth of 100 µm, the hardness stabilizes at values in the order of 280 HV. In the pearlite phase, this “softening” of the material is not noticed and the microhardness values are in the order of 300 to 320 HV. The greater the feed, the greater the microhardness value measured in both phases.

**Figure 15:**
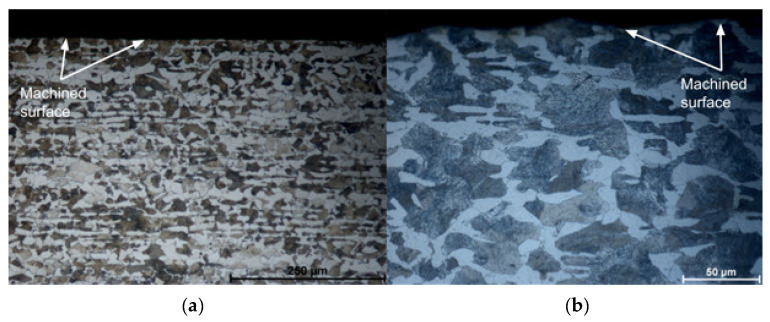
Microstructure of machined surfaces at *Vc* = 275 m/min: (**a**) *f* = 0.025 mm/rev (magnification 200×); (**b**) *f* = 0.1 mm/rev (magnification 500×).

When the cutting speed is increased to 275 m/min, in the pearlite phase this “softening” is observed just below the machined surface (25 µm deep) reaching hardness values in the order of 260 HV. Then, there is the stabilization of microhardness between 300 and 320 HV from a depth of 100 µm. In the ferrite phase, the microhardness values hardly vary in the depths observed for this cutting speed. Analyzing Figure 15, it is possible to observe that there is no microstructural alteration in the regions just below the machined surface. The grains remain relatively the same shape and size with a clear phase definition as seen in the core of the material. Although the cut is performed dry, the temperatures, as shown by the FEA analysis, do not reach the level for austenitizing of the material and the relatively low cutting forces, achieved due to the low values of feed and depth of cut, can contribute to this slight “softening” of the material just below the machined surface. Indeed, these results are not in agreement with the one established by Maruda et al. [9], who observed hardening of the ferrite and perlite phases just below the machined surface in the dry turning of 1045 steel, in addition to the presence of microcracks and deformations and the formation of a crumple zone. Certainly, the higher values used for feed (0.3 mm/rev) and depth of cut (2 mm), in addition to the use of coated carbide inserts, contributed to these factors, once the greatest values of cutting forces and temperatures in the cutting zone may have been reached.

## 4. Discussion

In the present work, AISI 1045 steel was dry turned with uncoated cermet inserts. The feed and cutting speed were varied.

The results of the FEA analysis showed good agreement regarding the shape and dimensions of the chips obtained. The chips were in the form of twisted ribbons of continuous type with their thickness varying as a function of the feed used. The good agreement of the chips obtained with those estimated by FEA analysis, in terms of shape and thickness, shows that this technique has good reliability for the results presented and discussed.

It was possible to verify through these analyses that the temperature in the contact zone between the tool and part does not exceed 860 °C, even for the highest cutting speed configuration used in the tests. Much of the heat generated in the cutting zone is expelled with the chip, thus also reducing the heat concentrated on the tool rake face. The cutting forces did not exceed 120 N, mainly due to the low cutting sections used. The tool geometry was also important in this regard.

As it is obvious by geometric and process kinematics, feed is a key factor in the roughness of the turned surface, while the cutting speed had little influence in this regard. This possibly happens due to the condition of the machined material, since, for example, it is known that when turning hardened materials (above 45 HRC) it is common for the roughness to be improved with increasing cutting speed. *Sa* = 0.23 µm roughness values can be achieved using a feed rate of 0.025 mm/rev. For all parameters used in the tests, the roughness values did not exceed *Sa* = 0. 97 µm, when a new cutting edge was used. In all feed rates used, the obtained roughness values were near the predicted theoretical roughness. When the feed is 0.075 mm/rev or 0.1 mm/rev, the surface roughness is less than that predicted by the theoretical equation. For a feed of 0.025 mm/rev, the theoretical roughness is 0.39 µm and the profile maximum height is 0.72 µm; for a feed of 0.05 mm/rev, the theoretical roughness is 1.57 µm and the profile maximum height is 1.77 µm; for a feed of 0.075 mm/rev, the theoretical roughness is 3.52 µm and the profile maximum height is 2.49 µm; and for a feed of 0.1 mm/rev, the theoretical roughness is 6.25 µm and the profile maximum height is 3.26 µm when the cutting speed is 175 m/min. If the *Ra* parameter is considered, as the feed increases, the experimental values are lower than the theoretical prediction as can be seen in Figure 9. 

With the 3D images obtained by interferometry, it is possible to observe that there were no defects caused by a possible poor formation of chips on the machined surfaces since these could be placed in a position adjacent to the edge of the insert coming into contact with the newly machined surface.

As expected, with the evolution of flank wear, the roughness is impaired. When the average flank wear *VB_B_* reaches 0.1 mm, a roughness of *Ra* = 2.92 µm is obtained, for a cutting speed of 275 m/min. The theoretical roughness for the feed of 0.025 mm/rev and corner radius of 0.2 mm is *Ra* = 0.39 µm. This factor is also strongly influenced by the notch wear observed on the main and secondary flanks of the insert. It can be said that these results are quite satisfactory when compared with other works in the literature such as Grzesik [13], notably because in the present work lower values of feed were used. The surface finish level for a new cutting edge can be compared with the results found by Sampaio et al. [3], under dry or MQL conditions. Cermet insert life is 35 min for *Vc* = 175 m/min and 23 min for *Vc* = 275 m/min when dry cutting is used, considering the end-of-life criterion *VB_B_* = 0.1 mm.

No increase in the microhardness of the machined surface or just below it was observed. On the contrary, a certain “softening” of the material was observed in the vicinity of the machined surface. The microhardness varies between 260–310 HV in the pearlite phase and 180–250 HV in the ferrite phase at 20 µm from the machined surface. No microstructure defects were observed, as well as crack propagation or accentuated deformations near the machined surface region. These results are at odds with those established by Maruda et al. [9], certainly due to the larger cross-sections used in the aforementioned work.

In this context, it is reasonable to say that the route of dry turning 1045 steel with cermet inserts is quite viable, since, with the appropriate parameters, a super-finished surface can be obtained without damaging the microstructure of the machined region. The life of the inserts can be considered satisfactory since the range of cutting speeds used in this research is in the highest range recommended by its manufacturer.

In future works [26], it is intended to use the MQL concept and cryogenics, as well as to use coated cermets to compare the results that have already been described here.

## 5. Conclusions

The conclusions drawn from the work are listed below:

- According to FEA analysis, the temperature in the cutting zone reaches 860 °C for the highest feed and cutting speed setting used in the experiments. The thickness and shape of the chips predicted by the FEA analysis were very close to the chips obtained in the experiments. The cutting forces did not exceed 120 N by the FEA analysis, mainly due to the small cut sections used.
- Feed is a key factor in surface roughness while cutting speed showed little influence on this aspect. Values in the order of *Sa* = 0.23 µm can be obtained when using the lowest values of feeds for a new cutting edge.
- As expected, the increase in flank wear impairs the surface roughness. When the wear reaches *VB*_B_ = 0.1 mm, the roughness is *Ra* = 2.92 µm. The notch wear on the primary and secondary flanks is decisive for this fact. Abrasive wear is predominant.
- A reduction in the microhardness values was observed, close to the machined surface (up to 100 µm in-depth) both in the pearlite and ferrite phases. No defects, cracks, deformations, or changes in the shape of the grains in the microstructure were observed, near the machined surface, within the cutting parameters used.
- It is noteworthy that the route of dry turning of 1045 steel, with uncoated cermet inserts, proved to be quite viable from the point of view of surface quality, tool life, microstructure, chip morphology, and sustainability.

## Figures and Tables

**Table 1:** Material properties.

Properties	Values
Tensile strength (MPa)	793
Yield strength (MPa)	718
Hardness (HB)	248

**Table 2:** Test cutting parameters.

Vc (m/min)	f (mm/rev)	Doc (mm)
175	0.025	0.2
	0.05	
	0.075	
	0.1	
275	0.025	
	0.05	
	0.075	
	0.1	

**Table 3:** Comparison between FEA analysis and optical measurement chip thickness.

Feed, *f* (mm/rev)	Cutting Speed, *Vc* (m/min)	Chip Thickness (mm)	
		FEA Analysis (σ)	Optical Measurement (σ)
0.025	175	0.043 (0.003)	0.045 (0.002)
	275	0.035 (0.001)	0.028 (0.002)
0.1	175	0.15 (0.02)	0.12 (0.03)
	275	0.17 (0.01)	0.18 (0.01)

## Data Availability

All data are available in the manuscript.

## References

[1] Jerold B.D., Kumar M.P. (2011). Experimental investigation of turning AISI 1045 steel using cryogenic carbon dioxide as the cutting fluid. J. Manuf. Process..

[2] Klocke F., Eisenblatter G. (1997). Dry Cutting. Ann. CIRP.

[3] Sampaio M.A., Machado R., Laurindo C.A.H., Torres R., Amorim F.L. (2018). Influence of minimum quantity of lubrication (MQL) when turning hardened SAE 1045 steel: A comparison with dry machining. Int. J. Adv. Manuf. Technol..

[4] Peng Y., Miao H., Peng Z. (2013). Development of TiCN-based cermets: Mechanical properties and wear mechanism. Int. J. Refract. Met. Hard Mater..

[5] Klocke F. (2011). Manufacturing Processes 1: Cutting.

[6] Ettmayer P., Kolaska H., Lengauer H., Dreyer K. (1995). Ti (C, N) cermets—Metallurgy and properties. Int. J. Refract. Met. Hard. Mater..

[7] Chen X., Xu J., Xiao Q. (2015). Cutting performance and wear characteristics of Ti(C,N)-based cermet tool in machining hardened steel. Int. J. Refract. Met. Hard Mater..

[8] Reis B.C., dos Santos A.J., dos Santos N.P., Câmara M.A., da Faria P.E., Abrão A.M. (2019). Cutting performance and wear behavior of coated cermet and coated carbide tools when turning AISI 4340 steel. Int. J. Adv. Manuf. Technol..

[9] Maruda R., Krolczyk G.M., Michalski M., Nieslony P., Wojciechowski S. (2017). Structural and Microhardness Changes After Turning of the AISI 1045 Steel for Minimum Quantity Cooling Lubrication. J. Mater. Eng. Perform..

[10] Sarjana S., Bencheikh I., Nouari M., Ginting A. (2020). Study on cutting performance of cermet tool in turning of hardened alloy steel. Int. J. Refract. Met. Hard Mater..

[11] Yang T., Ni L., Xiong J., Shi R., Zheng Q. (2018). Flank wear mechanism and tool endurance of (Ti,W)C-Mo2C-Co cermets during dry turning. Ceram. Int..

[12] Das A., Mukhopadhyay A., Patel S.K., Biswal B.B. (2016). Comparative Assessment on Machinability Aspects of AISI 4340 Alloy Steel Using Uncoated Carbide and Coated Cermet Inserts During Hard Turning. Arab. J. Sci. Eng..

[13] Grzesik W. (2008). Influence of tool wear on surface roughness in hard turning using differently shaped ceramic tools. Wear.

[14] Abbas F., Benyahia M., Rayes C., Pruncu C., Taha M., Hegab H. (2019). Towards Optimization of Machining Performance and Sustainability Aspects when Turning AISI 1045 Steel under Di. Materials.

[15] Magalhães L.C., da Silva Martins P.D., Souza M.T., Sombra S.C. Avaliação do desgaste de flanco em insertos de cermet no torneamento do aço ABNT 1020. Proceedings of the XI Brazilian Conference on Manufacturing Engineering.

[16] Magalhães L.C., Siqueira L., Silva W., Torres A. The influence of feed and tool nose radius on surface roughness in AISI 4340 high speed turning. Proceedings of the COBEF ANNALS.

[17] Zhang X.P., Wu S.B. (2017). Chip control in the dry machining of hardened AISI 1045 steel. Int. J. Adv. Manuf. Technol..

[18] Kumar C.S., Zeman P., Polcar T. (2020). A 2D finite element approach for predicting the machining performance of nanolayered TiAlCrN coating on WC-Co cutting tool during dry turning of AISI 1045 steel. Ceram. Int..

[19] Amigo F.J., Urbikain G., Pereira O., Fernández-Lucio P., Fernández-Valdivielso A., de Lacalle L.L. (2020). Combination of high feed turning with cryogenic cooling on Haynes 263 and Inconel 718 superalloys. J. Manuf. Process..

[20] Suárez L., López de Lacalle R., Polvoroza F., Veiga A., Wretland A. (2017). Effects of high-pressure cooling on the wear patterns on turning inserts used on alloy IN718. Mater. Manuf. Process.

[21] ISO (1996). ISO 4288: Geometrical Product Specification (GPS)—Surface Texture: Profile Method—Rules and Procedures for the Assessment of Surface Texture.

[22] Shaw M.C. (2004). Metal Cutting Principles.

[23] ISO (1993). ISO 3685—Tool Life Testing Wit Single Point Turning Tools.

[24] Brown C.A. (2017). Roughness Measurement Guidebook: Introduction to Surface Roughness Measurement.

[25] Davim J. (2010). Surface Integrity in Machining.

[26] Pereira O., Rodríguez A., Calleja-Ochoa A., Celaya A., de Lacalle L.N.L., Fernández-Valdivielso A., González H. (2022). Simulation of Cryo-cooling to Improve Super Alloys Cutting Tools. Int. J. Precis. Eng. Manuf. Technol..

